# Effects of an Antibiotic Alternative Additive on Growth Performance, Intestinal Histomorphology, Multi-Omics Profiles, and Microbiome–Host Interactions in Tibetan Pigs

**DOI:** 10.3390/vetsci13080826

**Published:** 2026-08-18

**Authors:** Zhaolong Li, Hongyang Zhao, Peimin Li, Song Peng, Junru Mo, Guiheng Mei, Meiqin Li, Fengqiang Lin, Quanwang Wu, Dexicuomu, Danzengzhuoga, Sangjiebazhu, Pubuzhaxi

**Affiliations:** 1 Institute of Animal Husbandry and Veterinary Medicine, Fujian Academy of Agricultural Sciences, Fuzhou 350013, China; pengsong@faas.cn (S.P.); m2981402250@163.com (J.M.); 18738371395@163.com (G.M.); limky02237@gmail.com (M.L.); linfengqiang@faas.cn (F.L.); 2 Changdu Animal Husbandry General Station, Changdu 854000, China18108958239@163.com (P.L.); wqw20000616@163.com (Q.W.); 13322552288@163.com (D.); 13618950665@163.com (D.); 271763028@163.com (S.); changdouxumu@sina.com (P.)

**Keywords:** Tibetan pig, antibiotic alternative, growth performance, intestinal histology, gut microbiome, metabolomics, multi-omics integration

## Abstract

The ban on antibiotic growth promoters has created an urgent need for effective alternatives to maintain intestinal health and production performance. This exploratory study evaluated the effects of a novel antibiotic alternative additive at graded inclusion levels (control, 1%, 3%, 5%) on growth performance, intestinal histomorphology, gut microbiome ecology, and host metabolome in Tibetan pigs (*n* = 40). Forty Tibetan pigs were randomly assigned to four dietary treatment groups for a 21-day feeding trial. Growth performance, intestinal histopathology, metatranscriptomic sequencing (bacteria, viruses, fungi), and untargeted metabolomics were analyzed. Tibetan pigs fed the 3% additive diet exhibited the highest numerical average daily gain (0.197 vs. 0.148 kg/d, +33.1%) and lowest feed conversion ratio (2.61 vs. 3.59, −27.3%), with overall treatment effects reaching statistical significance (ADG: *p* = 0.027; FCR: *p* = 0.023). Metatranscriptomic profiling revealed fundamentally distinct microbial communities between the duodenum and colon. Multi-omics integration identified 79 significant microbiome-pathology associations in the duodenum and 38 in the colon, with SCFA-producing bacteria positively correlated with mucosal architectural parameters. The multi-omics network revealed a core-periphery architecture with *Eubacterium*, *Lactobacillus*, and *Escherichia* as keystone hubs. Dietary supplementation at 3% showed the most favorable effects on growth performance and metabolic modulation without compromising intestinal structural integrity, supporting its potential as a viable antibiotic alternative.

## Simple Summary

The overuse of antibiotics in livestock production has raised serious concerns regarding antimicrobial resistance and food safety. Antibiotic alternatives that can maintain animal health and growth performance are urgently needed. In this study, we evaluated the effects of a novel antibiotic alternative additive (a probiotic-fermented soybean meal and corn-based compound) supplemented at control (CN, basal diet), 1%, 3%, and 5% in the diet of Tibetan pigs over a 21-day feeding trial. We comprehensively assessed growth performance, intestinal histomorphology, gut microbiome composition (bacteria, viruses, and fungi), and the metabolome. Our results showed that the 3% supplementation level improved average daily gain by 33.1% (overall ANOVA *p* = 0.027) and reduced the feed conversion ratio by 27.3% (overall ANOVA *p* = 0.023) compared to the control. Metatranscriptomic profiling revealed that the duodenum and colon harbor fundamentally distinct microbial communities: the duodenum was dominated by probiotic-associated genera (*Lactobacillus*, *Bifidobacterium*, *Streptococcus*, *Enterococcus*), whereas the colon was enriched in obligate anaerobic, fiber-degrading, short-chain fatty acid (SCFA)-producing taxa (*Bacteroides*, *Faecalibacterium*, *Prevotella*, *Ruminococcus*, *Roseburia*, *Clostridium*, *Eubacterium*, *Akkermansia*). Multi-omics integration revealed that beneficial SCFA-producing bacteria were strongly associated with healthy intestinal mucosal architecture, while opportunistic pathogens and specific *bacteriophages* were linked to inflammatory indices. These findings provide exploratory multi-omics-level evidence supporting the potential of this additive as an antibiotic alternative in Tibetan pig production.

## 1. Introduction

The Tibetan pig (*Sus scrofa domesticus*) is an indigenous breed native to the Qinghai–Tibet Plateau, valued for its adaptation to high-altitude, low-oxygen environments and its superior meat quality characterized by high intramuscular fat content and favorable fatty acid profiles [1,2,3]. However, Tibetan pigs typically exhibit slower growth rates (ADG approximately 0.10–0.15 kg/d under traditional management) and lower feed efficiency compared to commercial hybrid breeds, which poses challenges for intensive production systems [4]. Antibiotics have historically been used as growth promoters to enhance feed efficiency and control enteric diseases in swine production [5]. However, the global emergence of antimicrobial resistance and the subsequent ban on antibiotic growth promoters in many countries, including China’s comprehensive ban in 2020, have driven an urgent need for safe and effective alternatives [6,7].

Antibiotic alternatives encompass a diverse range of products, including probiotics, prebiotics, organic acids, phytogenic compounds, enzymes, and antimicrobial peptides, each acting through distinct mechanisms to modulate the gut ecosystem and promote host health [8,9]. Probiotic-fermented feed ingredients can improve gut health through multiple mechanisms, including competitive exclusion of pathogens, production of antimicrobial metabolites, enhancement of intestinal barrier function, and immunomodulation [9]. Fermented soybean meal, in particular, has been shown to reduce anti-nutritional factors, increase protein digestibility, and promote beneficial gut microbiota [9]. The intestinal tract harbors a complex microbial community comprising bacteria, archaea, viruses (including bacteriophages), and fungi, collectively termed the gut microbiome, which plays a fundamental role in nutrient digestion, immune regulation, barrier function, and overall host physiology [10,11]. The virome and mycobiome, although less studied than the bacteriome, are increasingly recognized as integral components of the gut ecosystem that influence host–microbe interactions [12,13]. Dietary interventions, particularly those involving fiber-rich feed additives, can profoundly reshape the composition and function of the gut microbiome, thereby modulating host metabolic and immune phenotypes through microbial metabolites such as short-chain fatty acids (SCFAs), bile acids, and tryptophan derivatives [14,15].

Recent advances in multi-omics technologies, including metatranscriptomics, metabolomics, and computational histopathology, now permit a systems-level understanding of how feed additives influence the complex interplay among the gut microbiome, host tissue architecture, and systemic metabolism [16,17]. However, comprehensive multi-omics studies evaluating antibiotic alternatives in Tibetan pigs, particularly those integrating bacteriome, virome, mycobiome, metabolome, and quantitative histopathology, remain scarce.

In the present study, we conducted a 21-day feeding trial in which Tibetan pigs received a novel antibiotic alternative additive (a compound probiotic-fermented soybean meal and corn-based product containing Lactobacillus spp., Bacillus spp., and their fermentation metabolites, including organic acids, enzymes, and antimicrobial peptides) at four dietary inclusion levels (CN, 1%, 3%, and 5%). We employed an integrative analytical framework combining growth performance metrics, intestinal histomorphological quantification (H&E and AB-PAS staining), metatranscriptomic profiling of bacteria, viruses, and fungi from duodenal and colonic luminal contents, untargeted metabolomics, and multi-omics correlation network analysis. We hypothesized that the 3% additive inclusion level would optimally balance growth performance and metabolic modulation without compromising intestinal mucosal integrity.

## 2. Materials and Methods

### 2.1. Animals, Experimental Design, and Diets

The feeding trial was conducted at a commercial pig farm (Dovuka Breeding Pig Farm, Changdu City, Tibet Autonomous Region, China) from 4 December to 25 December 2025, over a 21-day experimental period. Forty healthy Tibetan pigs (mixed sex, 95 days old) with an average initial body weight of 8.26 ± 0.34 kg were randomly assigned to four dietary treatment groups: a CN (control) group receiving a conventional basal diet (Table S1) without the additive (*n* = 10); a 1% group receiving the basal diet supplemented with 1% (*w*/*w*, replacing an equivalent proportion of the basal diet) antibiotic alternative additive (*n* = 10); a 3% group receiving the basal diet supplemented with 3% additive (*n* = 10); and a 5% group receiving the basal diet supplemented with 5% additive (*n* = 10). Feed was provided ad libitum, with each group receiving approximately 100 kg of feed over the 21-day period (actual consumption varied by group). Pigs were housed in group pens (10 pigs per pen, one pen per treatment group) with concrete flooring under natural lighting and ambient temperature conditions. Feed was provided in group feeders, and water was available ad libitum via nipple drinkers. Health status was monitored daily by visual observation by a qualified veterinarian. Pigs were vaccinated against classical swine fever and foot-and-mouth disease according to the farm’s standard protocol. No antibiotics or other growth-promoting additives were administered during the trial period. The antibiotic alternative additive is a compound probiotic-fermented product manufactured by solid-state fermentation of soybean meal and corn (3:1 ratio) using a mixed starter culture of *Lactobacillus plantarum* (≥1.0 × 10^9^ CFU/g), *Bacillus subtilis* (≥5.0 × 10^8^ CFU/g), and *Saccharomyces cerevisiae* (≥2.0 × 10^8^ CFU/g). The fermentation process (37 °C, 72 h) yields a product rich in organic acids (lactic acid, acetic acid), enzymes (protease, amylase, phytase), antimicrobial peptides, and partially hydrolyzed proteins. The final product contains ≥40% crude protein, ≤10% crude fiber, and ≥5% lactic acid on a dry matter basis. The three inclusion levels (1%, 3%, 5% *w*/*w*) were selected based on previous dose-finding studies in swine [8] and correspond to replacing an equivalent proportion of the basal diet.

### 2.2. Growth Performance Measurements

Individual body weight was recorded at the beginning (day 0) and at the end (day 21) of the trial. Feed intake per group was recorded daily. The following growth performance parameters were calculated: initial body weight (IBW), final body weight (FBW), average daily gain (ADG), average daily feed intake (ADFI), and feed conversion ratio (FCR = ADFI/ADG). Data are presented as mean ± SEM. Growth performance data were analyzed using one-way ANOVA with Tukey HSD post hoc test for pairwise comparisons among the four treatment groups. Statistical significance was declared at *p* < 0.05. Superscript letters (a, b, ab) in tables denote significant differences between groups. All statistical analyses were performed using SPSS 26.0 (IBM Corp., Armonk, NY, USA) and Python 3.x (scipy.stats).

### 2.3. Sample Collection

At the end of the 21-day feeding trial, all pigs were euthanized by a two-step procedure: electrical stunning (head-only, 1.3 A, 50 Hz, 3 s) to induce immediate loss of consciousness, followed by exsanguination via severance of the carotid arteries and jugular veins. Intestinal tissue segments (duodenum and jejunum) were collected from 3 randomly selected pigs per group (*n* = 3 per group, 12 pigs total; this sample size is consistent with an exploratory study design and was based on resource availability and ethical considerations for minimizing animal use for each intestinal segment) and immediately fixed in 10% neutral buffered formalin for histological analysis. Intestinal content samples from the duodenum and colon were collected from 12 pigs per site (*n* = 3 per group × 4 groups = 12 per site, 24 total) into sterile cryovials, snap-frozen in liquid nitrogen, and stored at −80 °C for metatranscriptomic and metabolomic analyses.

### 2.4. Histological Analysis

#### 2.4.1. H&E and AB-PAS Staining

Formalin-fixed intestinal tissues were dehydrated through a graded ethanol series, cleared in xylene, and embedded in paraffin. Tissue sections (4 μm thickness) were cut and stained with hematoxylin and eosin (H&E) for morphological assessment and with Alcian Blue–Periodic Acid Schiff (AB-PAS) for mucin and goblet cell visualization. Whole-slide panoramic images were acquired using a digital slide scanner at 40× magnification (Pannoramic 250 Flash III, 3DHISTECH Ltd., Budapest, Hungary). Tissue processing was performed using a Leica TP1020 automatic tissue processor (Leica Biosystems, Nussloch, Germany). For H&E staining, sections were deparaffinized in xylene, rehydrated through graded ethanol, stained with Harris hematoxylin (5 min), differentiated in 1% acid alcohol, blued in Scott’s tap water, counterstained with 1% eosin Y (2 min), dehydrated, cleared, and mounted with DPX. For AB-PAS staining, sections were stained with Alcian Blue (pH 2.5, 30 min), oxidized in 1% periodic acid (10 min), treated with Schiff’s reagent (15 min), counterstained with hematoxylin, dehydrated, cleared, and mounted, embedding was performed on a Leica EG1150 H paraffin embedding station, and sectioning was performed on a Leica RM2255 rotary microtome (Leica Biosystems, Nussloch, Germany). For H&E staining, sections were deparaffinized in xylene, rehydrated through graded ethanol, stained with Harris hematoxylin (5 min), differentiated in 1% acid alcohol, blued in Scott’s tap water, counterstained with 1% eosin Y (2 min), dehydrated, cleared, and mounted with DPX. For AB-PAS staining, sections were stained with Alcian Blue (pH 2.5, 30 min), oxidized in 1% periodic acid (10 min), treated with Schiff’s reagent (15 min), counterstained with hematoxylin, dehydrated, cleared, and mounted. H&E and AB-PAS staining were performed using a Leica ST5020 automatic staining machine (Leica Biosystems, Nussloch, Germany). For H&E staining, sections were deparaffinized in xylene, rehydrated through graded ethanol, stained with Harris hematoxylin (5 min), differentiated in 1% acid alcohol, blued in Scott’s tap water, counterstained with 1% eosin Y (2 min), dehydrated, cleared, and mounted with DPX. For AB-PAS staining, sections were stained with Alcian Blue (pH 2.5, 30 min), oxidized in 1% periodic acid (10 min), treated with Schiff’s reagent (15 min), counterstained with hematoxylin, dehydrated, cleared, and mounted.

#### 2.4.2. Computational Histopathological Quantification

All histological measurements were performed in a blinded manner. Slide identities were anonymized by an independent researcher before image acquisition and quantitative analysis. Intra-observer variability was assessed by re-measuring 20% of randomly selected samples; the intra-class correlation coefficient (ICC) exceeded 0.90 for all quantitative metrics.

Quantitative image analysis was performed using a custom Python 3.x pipeline incorporating OpenCV, scikit-image, and SciPy libraries [18]. Tissue segmentation was achieved by a three-channel voting method combining Otsu thresholding, HSV saturation thresholding (>12), and LAB luminosity thresholding (<235), followed by morphological closing (20 × 20 px) and opening (15 × 15 px) operations with hole-filling, retaining the largest connected component as the tissue mask.

For H&E analysis, color deconvolution was performed using the standard hematoxylin/eosin/residual stain matrix following Ruifrok and Johnston (2001) [19]. Villus height and crypt depth were quantified using a 12-column sliding window vertical profile analysis, with the villus–crypt junction identified as the valley of the tissue density profile; values were normalized to image height. Muscle thickness was determined by eosin-positive pixels exceeding the mean + SD threshold in the lower 30% of the tissue, normalized to image height. Pathology scoring (0–3 for each parameter) included inflammation score (based on nuclear density), epithelial damage score (edge integrity assessment), and edema score (void ratio); total pathology score ranged from 0 to 9.

For AB-PAS analysis, Alcian Blue-positive (acidic mucin, AB+) pixels were detected by a three-channel voting algorithm combining LAB B/A channels, HSV H/S/V channels, and RGB criteria (B > R × 1.03 and B > G × 1.03). PAS-positive (neutral mucin) pixels were detected using R/G and R/B ratios, HSV hue range for magenta, and the LAB A channel. Goblet cells were identified by connected-component analysis on the blue/purple mask filtered by area (10–600 px^2^), circularity (>0.15), or solidity (>0.3); density was expressed per mm^2^. Mucin percentages were quantified as positive pixels normalized to tissue mask area (×100%).

#### 2.4.3. Statistical Analysis of Histological Data

The Kruskal–Wallis nonparametric rank-sum test was used to assess overall group effects across the four treatment groups. Post hoc pairwise comparisons were performed using two-sided Mann–Whitney U tests with Bonferroni correction for 6 comparisons per metric. Significance levels were defined as: † *p* < 0.10 (trend), * *p* < 0.05, ** *p* < 0.01, and *** *p* < 0.001. With *n* = 3 per group, the minimum achievable two-sided Mann–Whitney U *p*-value was 0.10 (U = 0); therefore, trend-level significance († *p* < 0.10) is reported.

### 2.5. Metatranscriptomic Sequencing and Analysis

Total RNA for metatranscriptomic analysis was extracted from duodenal (S) and colonic (J) content samples (*n* = 3 per group per site, 24 samples total) using a commercial RNA extraction kit (RNeasy Mini Kit, Qiagen, Hilden, Germany) following the manufacturer’s protocol. RNA concentration and integrity were assessed by Qubit fluorometry (RNA HS Assay Kit, Thermo Fisher Scientific, Waltham, MA, USA) and agarose gel electrophoresis. Metatranscriptomic libraries were prepared using the MGI Easy RNA Library Prep Kit, MGI Tech Co., Ltd., Shenzhen, China and sequenced on the MGI DNBSEQ-T7 platform, MGI Tech Co., Ltd., Shenzhen, China (paired-end 150 bp). Raw reads were quality-filtered using fastp [20], and host-derived reads were removed by mapping against the Sus scrofa reference genome (Sscrofa11.1) using Bowtie2 [21]. Taxonomic classification of microbial reads was performed using Kraken2 [22] against a standard RefSeq database (release 2024; encompassing bacterial, viral, and fungal genomes). The mean sequencing depth was 15.2 ± 3.4 million paired-end reads per sample. Host-derived reads were filtered by mapping against the *Sus scrofa* reference genome (Sscrofa11.1) using Bowtie2 with default parameters, removing on average 12.3% of total reads. Contamination from laboratory reagents and environmental sources was controlled by including negative extraction controls and applying the decontam R package (prevalence method, threshold = 0.5). Relative abundances were computed at the genus level for bacteria and fungi and at the family level for viruses.

### 2.6. Untargeted Metabolomics

#### 2.6.1. Sample Preparation and Uhplc-Ms/Ms Analysis

Colon tissue samples (*n* = 3 per group, 12 total) were subjected to metabolite extraction using a methanol:acetonitrile:water (2:2:1, *v*/*v*/*v*) solvent system. After vortexing, sonication, and centrifugation, the supernatant was collected and dried under vacuum. Dried extracts were reconstituted in 50% acetonitrile. Non-targeted metabolomic profiling was performed on an ultra-high-performance liquid chromatography–tandem mass spectrometry (UHPLC-MS/MS) system coupled to a tandem mass spectrometer (MS/MS) operating in both positive (POS) and negative (NEG) electrospray ionization modes. Chromatographic separation was achieved using a C18 reversed-phase column with a binary gradient of water (0.1% formic acid) and acetonitrile (0.1% formic acid). Full-scan MS and data-dependent MS/MS spectra were acquired over a mass range of *m*/*z* 50–1500 at a resolution of 70,000 (MS) and 17,500 (MS/MS).

#### 2.6.2. Data Processing and Statistical Analysis

Raw MS data were processed for peak detection, alignment, and integration. Metabolite identification was performed by matching accurate mass, isotope patterns, and MS/MS fragmentation spectra against public databases (HMDB, METLIN, and MassBank), as well as an in-house authentic standard library. Metabolites were classified according to the HMDB superclass/class/subclass taxonomy.

Principal component analysis (PCA) was performed on log_2_(x + 1)-transformed and Z-score standardized metabolite abundances. Multi-group differential metabolite screening was conducted using one-way ANOVA across the four groups, with pairwise comparisons (1% vs. CN, 3% vs. CN, 5% vs. CN) performed using Student’s *t*-test. *p*-values were adjusted for multiple testing using the Benjamini–Hochberg false discovery rate (FDR) procedure. Metabolites were considered significantly differential at FDR < 0.05. Fold change (FC) was calculated as the ratio of mean abundances between groups. KEGG pathway enrichment analysis was performed using a hypergeometric test with Benjamini–Hochberg FDR correction.

### 2.7. Multi-Omics Integrative Correlation Analysis

Given the exploratory nature of this study, a formal a priori power calculation was not performed. The sample size for multi-omics analyses (*n* = 3 per group) was determined by resource availability and ethical considerations. With *n* = 3 per group, the minimum achievable two-sided Mann–Whitney U *p*-value is 0.10 (U = 0); therefore, trend-level significance (*p* < 0.10) is reported for histology and omics analyses, and all results from these analyses should be interpreted as exploratory. The implications of limited statistical power for Type II error are addressed in the Discussion.

All samples within each analytical modality (metatranscriptomics, metabolomics) were processed in a single batch to minimize batch effects. Spearman rank correlation analysis was performed between microbial taxa abundances and histopathological metrics at the treatment-group level (*n* = 4 groups per intestinal site: CN, 1%, 3%, 5%) for the duodenum (S) and colon (J) separately. Significance thresholds for microbiome–pathology associations were set at FDR < 0.3 and |ρ| > 0.4. For microbiome–metabolome associations, the more stringent threshold of |ρ| > 0.5 was applied without additional FDR correction, given the larger dimensionality of the metabolomics dataset and the exploratory nature of this analysis. In the duodenum (S site), 8817 differential metabolites were screened at a relaxed threshold (FDR < 0.1) to capture broader metabolic variation for exploratory correlation analysis; the higher number compared to the colon (2180 at FDR < 0.05) reflects both the more permissive FDR cutoff and the distinct metabolic activity of the two intestinal segments. Benjamini–Hochberg FDR correction was applied across all pairwise tests. Multi-omics interaction networks were visualized using a spring layout algorithm with edge width proportional to |ρ|. Red edges denoted positive correlations; blue edges denoted negative correlations. Network nodes were coded as circles (microbes), squares (pathology metrics), and diamonds (metabolites).

### 2.8. Software and Code Availability

All bioinformatic and statistical analyses were performed using Python 3.x with the following packages: pandas, numpy, scipy (v1.x), statsmodels, scikit-learn, matplotlib (v3.x), seaborn (v0.12+), networkx, OpenCV (cv2), and scikit-image. Figure assembly was performed using matplotlib GridSpec and Adobe Illustrator 28.5. All custom analysis scripts are available from the corresponding author upon reasonable request.

## 3. Results

### 3.1. Growth Performance

Table 1 summarizes the growth performance of Tibetan pigs across the four dietary treatment groups. Initial body weights were not significantly different among groups (*p* = 0.347), confirming successful randomization. After the 21-day feeding period, the 3% additive group exhibited the highest numerical final body weight (12.85 ± 0.36 kg vs. 11.36 ± 0.58 kg in the CN group), the highest average daily gain (0.197 ± 0.017 kg/d vs. 0.148 ± 0.016 kg/d, +33.1%), and the lowest feed conversion ratio (2.61 ± 0.25 vs. 3.59 ± 0.39, −27.3%). The 1% and 5% groups showed intermediate improvements, with ADG values of 0.172 ± 0.022 and 0.179 ± 0.012 kg/d, and FCR values of 3.24 ± 0.43 and 3.05 ± 0.22, respectively. The average daily feed intake was comparable across the CN, 1%, and 3% groups (0.476 kg/d), with a slightly higher value in the 5% group (0.529 ± 0.031 kg/d).

### 3.2. Intestinal Histomorphology

#### 3.2.1. Jejunum Histology

**Figure 1:**
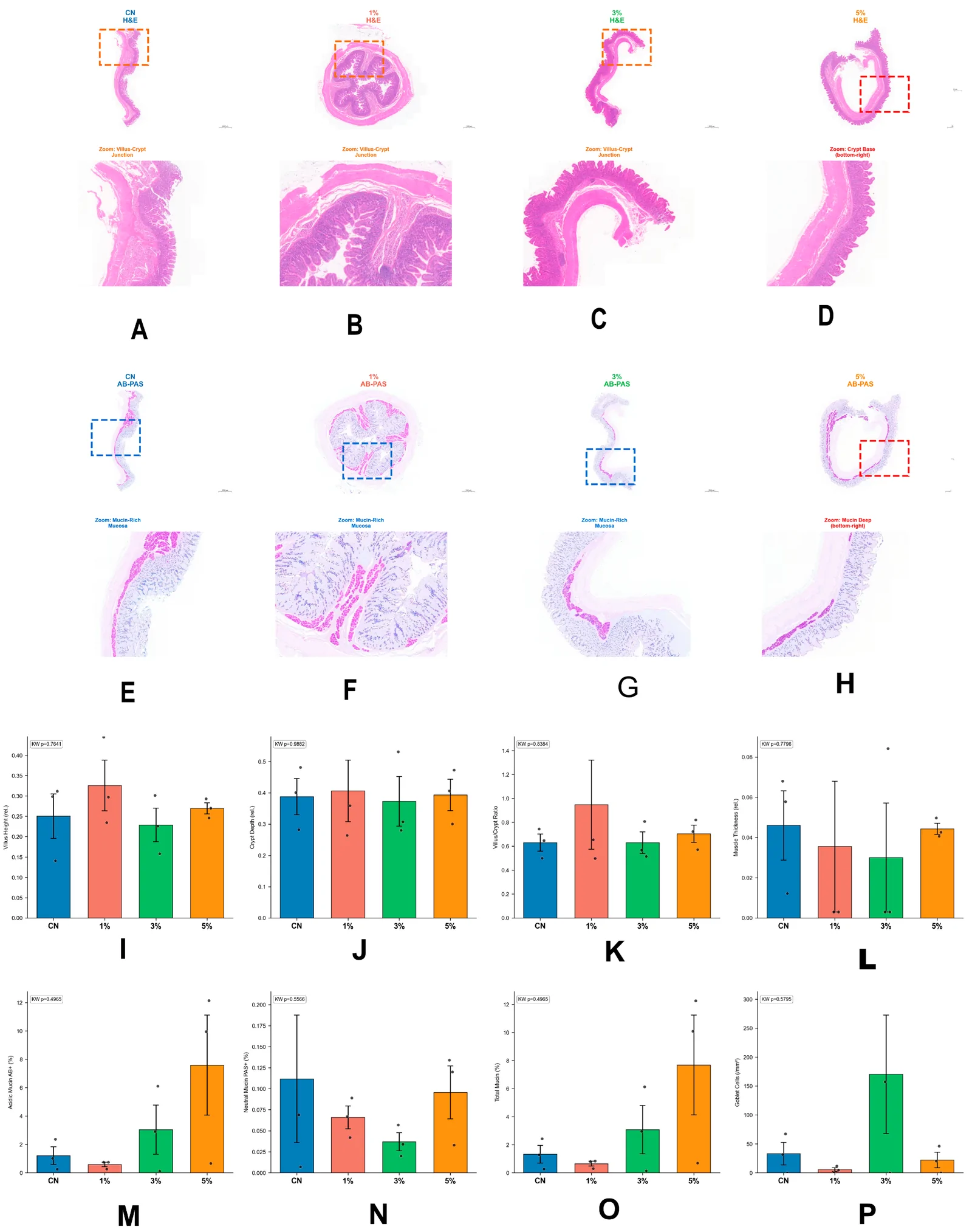
Representative histological analysis of jejunum tissue sections from Tibetan pigs across four treatment groups (CN = Control, 1% = Low dose, 3% = Medium dose, 5% = High dose) with H&E and AB-PAS staining. (**A**–**D**) H&E-stained whole-tissue panoramic images (CN, 1%, 3%, 5% groups, respectively). Dashed boxes indicate zoom-in regions: red dashed box (5% group) anchored at the bottom-right corner of the tissue section, focusing on the deep crypt region; orange dashed boxes (all other groups) automatically placed at the villus–crypt junction area. Scale bar = 500 μm. (**E**–**H**) H&E zoom-in views corresponding to boxed regions in (**A**–**D**). Scale bar = 100 μm. (**I**–**L**) AB-PAS-stained whole-tissue panoramic images (CN, 1%, 3%, 5% groups, respectively). Dashed boxes indicate zoom-in regions: red dashed box (5% group) at the bottom-right corner; blue dashed boxes (all other groups) at the mucin-rich mucosa region. Scale bar = 500 μm. (**M**–**P**) AB-PAS zoom-in views corresponding to boxed regions in (**I**–**L**). Scale bar = 100 μm. Metrics shown: villus height ratio, crypt depth ratio, villus/crypt (V/C) ratio, muscle thickness ratio, nuclear density, inflammation score, and total pathology score. Individual data points shown as black dots. Statistical analysis: Kruskal–Wallis nonparametric test followed by Bonferroni-corrected post hoc Mann–Whitney U tests for pairwise comparisons. Significance: ns (not significant) *p* ≥ 0.10, † *p* < 0.10 (trend), * *p* < 0.05, ** *p* < 0.01. All data are presented as mean ± SEM. *n* = 3 biological replicates per group.

Quantitative H&E morphometric analysis of the jejunum revealed no significant differences in villus height, crypt depth, villus-to-crypt (V/C) ratio, or muscle thickness across the four treatment groups (Kruskal–Wallis test, all *p* > 0.05; Table 2). Similarly, nuclear density, inflammation scores, and total pathology scores did not differ significantly among groups, indicating that the dietary additive did not induce significant structural alterations, although the small sample size (*n* = 3 per group) limits the statistical power to detect subtle histomorphological changes (Type II error risk) in the jejunal epithelium at any inclusion level (Figure 1).

AB-PAS staining of jejunal sections (Table 2) showed that acidic mucin (AB+) content was numerically highest in the CN group (7.82 ± 1.74% tissue area) compared to the treated groups (1.77–3.85%), although this difference did not reach statistical significance (KW *p* = 0.108). Total mucin percentage (AB+ plus PAS+) followed a similar pattern (KW *p* = 0.108). Goblet cell density in the jejunum showed a trend-level decrease from 660.9 ± 230.6 cells/mm^2^ in the CN group to 82.0–103.4 cells/mm^2^ in the treated groups (KW *p* = 0.086, trend).

#### 3.2.2. Duodenum Histology

**Figure 2:**
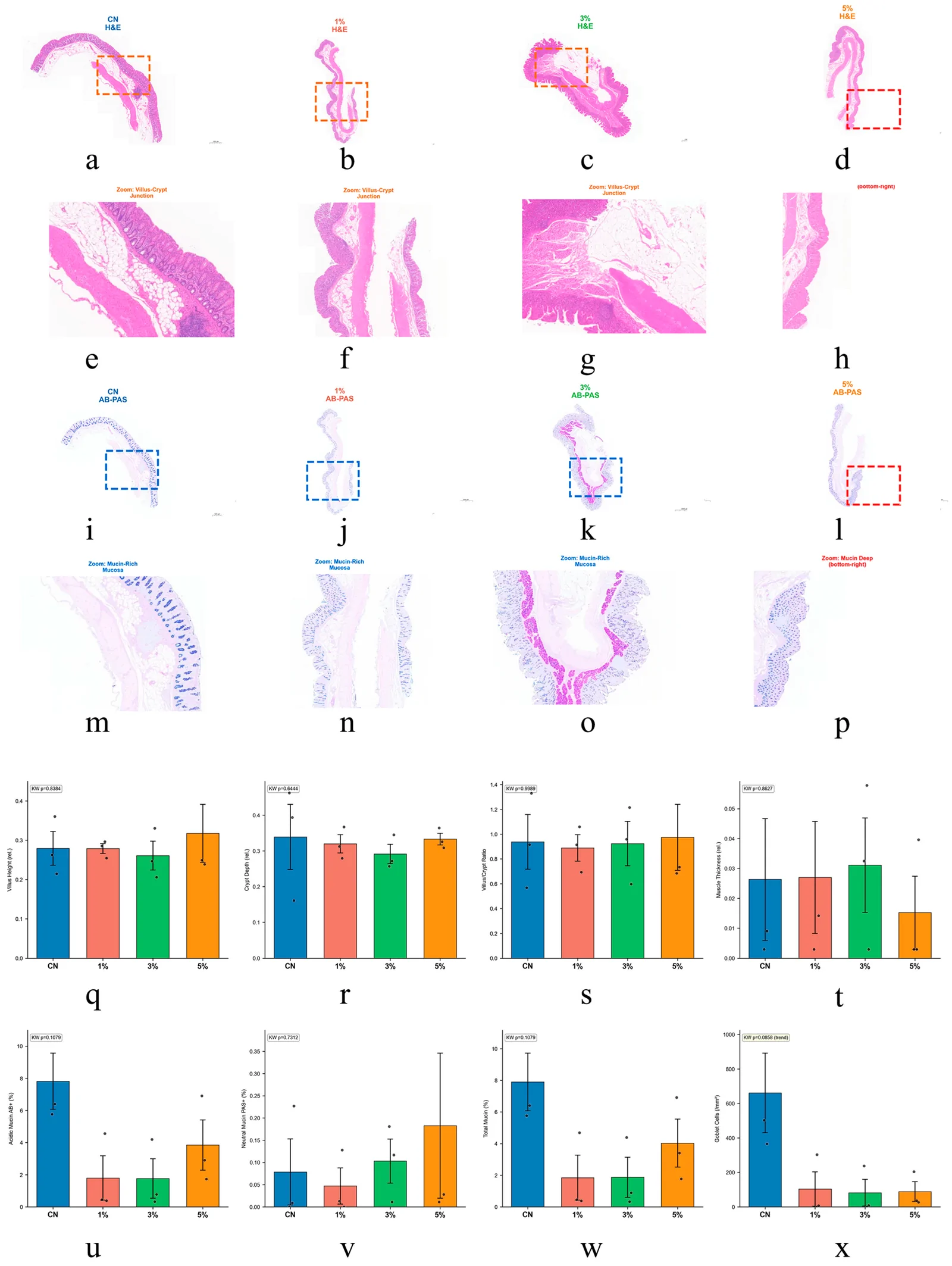
Representative histological analysis of duodenum tissue sections from Tibetan pigs across four treatment groups (CN = Control, 1% = Low dose, 3% = Medium dose, 5% = High dose) with H&E and AB-PAS staining. (**a**–**d**) H&E-stained whole-tissue panoramic images (CN, 1%, 3%, 5% groups, respectively). Dashed boxes indicate zoom-in regions: red dashed box (5% group) anchored at the bottom-right corner of the tissue section, focusing on the deep crypt region; orange dashed boxes (all other groups) automatically placed at the villus–crypt junction area. Scale bar = 500 μm. (**e**–**h**) H&E zoom-in views corresponding to boxed regions in (a–d). Scale bar = 100 μm. (**i**–**l**) AB-PAS-stained whole-tissue panoramic images (CN, 1%, 3%, 5% groups, respectively). Dashed boxes indicate zoom-in regions: red dashed box (5% group) at the bottom-right corner; blue dashed boxes (all other groups) at the mucin-rich mucosa region. Scale bar = 500 μm. (**m**–**p**) AB-PAS zoom-in views corresponding to boxed regions in (**i**–**l**). Scale bar = 100 μm. (**q**–**x**) Quantitative bar charts of H&E morphometric parameters (mean ± SEM, *n* = 3 biological replicates per group). Metrics shown: villus height ratio, crypt depth ratio, villus/crypt (V/C) ratio, muscle thickness ratio, nuclear density, inflammation score, and total pathology score. Individual data points shown as black dots. Statistical analysis: Kruskal–Wallis nonparametric test followed by Bonferroni-corrected post hoc Mann–Whitney U tests for pairwise comparisons. Significance: + *p* < 0.10 (trend), * *p* < 0.05, ** *p* < 0.01, *** *p* < 0.001. With *n* = 3 per group, the minimum achievable two-sided MWU *p*-value is 0.10; trend-level (+) significance is therefore reported where *p* < 0.10. No metrics reached *p* < 0.05 across groups. All data are presented as mean ± SEM. *n* = 3 biological replicates per group.

In the duodenum, H&E morphometric parameters were comparable across all treatment groups (Table 3). Villus height, crypt depth, V/C ratio, and muscle thickness showed no significant differences (all KW *p* > 0.05). Total pathology scores ranged from 3.67 to 5.33 across groups without significant inter-group differences (KW *p* = 0.122) (Figure 2).

AB-PAS analysis of the duodenum (Table 3) revealed a non-significant dose-dependent increasing trend in acidic mucin content from CN (1.21 ± 0.62%) to the 5% group (7.59 ± 3.52%; KW *p* = 0.497). Goblet cell density was highly variable, with a notable peak in the 3% group (170.2 ± 102.2 cells/mm^2^) compared to the CN (33.0 ± 19.4), 1% (5.4 ± 3.3), and 5% (22.1 ± 13.3) groups, though this difference was not statistically significant (KW *p* = 0.580). The acid-to-neutral mucin ratio showed a non-significant elevation in the 3% group (115.4 ± 89.8 vs. 23.7 ± 9.9 in CN; KW *p* = 0.361).

### 3.3. Gut Microbial Community Composition

**Figure 3:**
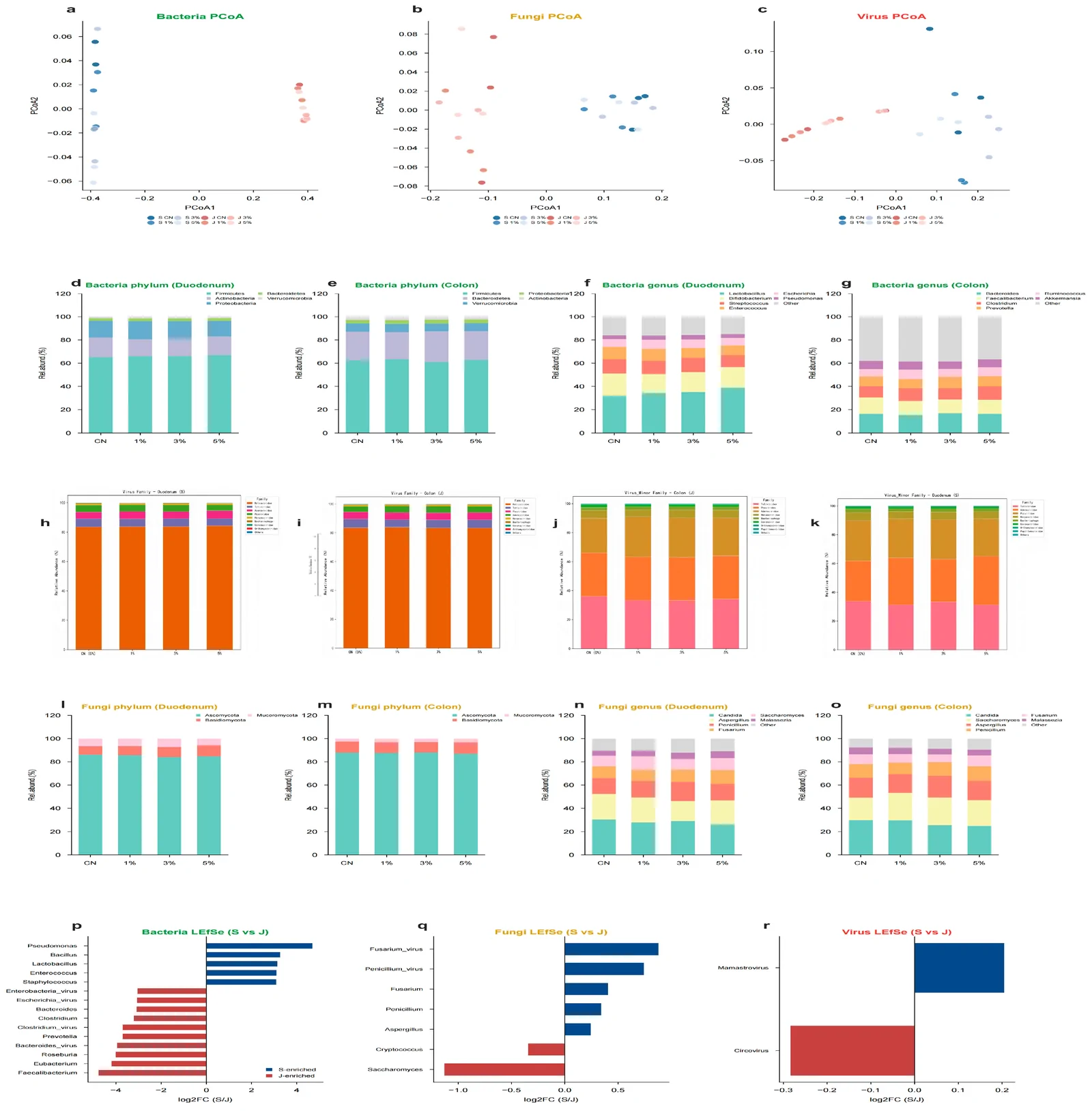
Gut Microbial Community Composition, Beta Diversity, and Site-Specific Biomarker Taxa in the Duodenum and Colon of Tibetan Pigs across Additive Dose Groups. (**a**–**c**) Principal coordinate analysis (PCoA) of Bray–Curtis distances showing beta diversity of (**a**) bacterial (genus level), (**b**) fungal (genus level), and (**c**) viral (family/genus level) communities in duodenal (S, diamonds) and colonic (J, circles) luminal contents (*n* = 12 per site, 24 samples total). Colors denote additive dose groups: CN (blue), 1% (orange), 3% (green), 5% (red). Ellipses represent 95% confidence intervals. S and J samples form clearly separated clusters across all three microbial kingdoms. (**d**–**g**) Stacked bar charts of bacterial community composition. (**d**) Phylum-level composition in duodenum (S) across doses. (**e**) Phylum-level composition in the colon (J) across doses. (**f**) Genus-level composition in duodenum (S). (**g**) Genus-level composition in the colon (J). (**h**–**k**) Stacked bar charts of viral community composition. (**h**) Phylum-level composition in duodenum (S). (**i**) Phylum-level composition in the colon (J). (**j**) Genus/family-level composition in duodenum (S). (**k**) Genus/family-level composition in colon (J). (**l**–**o**) Stacked bar charts of fungal community composition. (**l**) Phylum-level composition in duodenum (S). (**m**) Phylum-level composition in colon (J). (**n**) Genus-level composition in duodenum (S). (**o**) Genus-level composition in colon (J). (**p**–**r**) Linear discriminant analysis Effect Size (LEfSe) identifying tissue-discriminatory biomarker taxa between duodenum (S) and colon (J) (LDA score > 3.0, FDR < 0.05). (**p**) Bacterial LEfSe: 23 genera were significantly discriminated between S and J. S-enriched genera (positive LDA): *Lactobacillus*, *Bifidobacterium*, *Streptococcus*, *Enterococcus*, *Escherichia*, *Pseudomonas*, *Bacillus*, *Staphylococcus*, plus *Staphylococcus phage* (*Staphylococcus_virus*) and *Lactobacillus phage* (*Lactobacillus_virus*). J-enriched genera (negative LDA): *Bacteroides*, *Faecalibacterium*, *Prevotella*, *Ruminococcus*, *Roseburia*, *Clostridium*, *Eubacterium*, *Akkermansia*, plus *Escherichia phage* (*Escherichia_virus*), *Enterobacteria phage* (*Enterobacteria_virus*), *Bacteroides phage* (*Bacteroides_virus*), and *Clostridium phage* (*Clostridium_virus*). (**q**) Viral LEfSe: *Mamastrovirus* significantly enriched in S (log2FC = 0.20, FDR = 0.0014); *Circovirus* significantly enriched in J (log2FC = −0.28, FDR = 0.0014). (**r**) Fungal LEfSe: 7 genera significantly discriminated between sites. S-enriched: *Fusarium_virus*, *Penicillium_virus*, *Fusarium*, *Penicillium*, *Aspergillus*. J-enriched: *Saccharomyces*, *Cryptococcus*.

Metatranscriptomic sequencing of duodenal (S) and colonic (J) luminal content samples from 24 Tibetan pigs (*n* = 3 per tissue-dose group) revealed distinct microbial community structures across intestinal sites, with tissue origin constituting the dominant source of variation across all three microbial kingdoms (bacteria, viruses, and fungi; Figure 3). Beta diversity analysis using principal coordinate analysis (PCoA) of Bray–Curtis distances demonstrated clear separation between duodenal and colonic samples in the bacterial (Figure 3a), fungal (Figure 3b), and viral (Figure 3c) communities. The clustering by intestinal site consistently exceeded the variation attributable to additive dose (CN, 1%, 3%, 5%), indicating that anatomical location is the primary determinant of gut microbial community structure in Tibetan pigs.

#### 3.3.1. Bacterial Community Structure

Bacterial phylum-level profiling revealed that *Firmicutes* dominated both intestinal segments (J: 58–68%; S: ~68%), followed by *Bacteroidetes* (J: 20–29%), *Proteobacteria*, *Actinobacteria*, and *Verrucomicrobia* (Figure 3d,e). At the genus level, the duodenum and colon exhibited markedly distinct compositional profiles (Figure 3f,g). The duodenal bacteriome was characterized by high relative abundances of *Lactobacillus* (~35%), *Bifidobacterium* (~18%), *Streptococcus* (~12%), *Enterococcus* (~10%), and *Escherichia* (~7%), genera typically associated with small intestinal aerobic or facultative anaerobic niches. In contrast, the colon was enriched in obligate anaerobic and fiber-degrading taxa, including *Bacteroides* (~16%), *Faecalibacterium* (~12%), *Clostridium* (~11%), *Prevotella* (~9%), *Roseburia* (~6%), *Ruminococcus* (~7%), *Eubacterium* (~4%), and *Akkermansia* (~7%). This duodenal-colonic dichotomy—*Lactobacillaceae*-dominant in the small intestine versus *Bacteroidaceae-Ruminococcaceae*-dominant in the large intestine—is consistent with the known spatial organization of the porcine gut microbiome along the longitudinal axis and reflects the transition from aerobic digestion to anaerobic fermentation.

With increasing dietary additive supplementation (CN → 1% → 3% → 5%), the bacterial community composition of both intestinal segments exhibited gradual shifts; however, no dramatic restructuring of the overall community was observed at the phylum level, suggesting that the additive modulates relative abundances within the existing community framework rather than inducing wholesale taxonomic replacement.

#### 3.3.2. Viral Community Structure

The gut virome was dominated by two phyla, Pisuviricota and Cressdnaviricota, across both intestinal sites (Figure 3h,i). In the duodenum, Pisuviricota constituted 58–70% of classified viral sequences, with Cressdnaviricota accounting for the remaining 30–42%. At the genus/family level, Mamastrovirus predominated in the duodenum, whereas Circovirus showed elevated relative abundance in the colon (Figure 3j,k). Notably, the relative abundance of bacteriophages (particularly Staphylococcus phage, Lactobacillus phage, Enterobacteria phage, Bacteroides phage, and Clostridium phage) exhibited tissue-specific distribution patterns that paralleled their putative bacterial hosts, with phages targeting small-intestine-enriched genera (Staphylococcus_virus, Lactobacillus_virus) more abundant in the duodenum, and phages targeting colon-enriched genera (Escherichia_virus, Enterobacteria_virus, Bacteroides_virus, Clostridium_virus) more abundant in the colon, suggesting active phage-bacterium co-occurrence dynamics along the gastrointestinal tract [23].

#### 3.3.3. Fungal Community Structure

Fungal community diversity, though lower in taxonomic richness than the bacteriome, was characterized by three dominant phyla: *Ascomycota* (83–87%), *Basidiomycota* (7–11%), and *Mucoromycota* (5–8%) (Figure 3l,m). At the genus level, *Candida* was the most abundant fungal taxon across both sites (~27–28%), followed by *Aspergillus* (~17–20%) and *Penicillium* (~12–15%) (Figure 3n,o). Clear compositional differences were evident between the colon and duodenum: the duodenum harbored higher relative abundances of *Fusarium* (~11% vs. ~8%), *Penicillium* (~15% vs. ~12%), and *Aspergillus* (~20% vs. ~17%), while the colon was significantly enriched in *Saccharomyces* (~22% vs. ~10%) and *Cryptococcus* (~4% vs. ~3%). This site-specific mycobiome composition may reflect differential exposure to dietary and environmental fungi, as well as fungal-bacterial interactions that differ between the small and large intestinal environments.

#### 3.3.4. Site-Specific Biomarker Taxa

Linear discriminant analysis Effect Size (LEfSe) analysis (LDA score > 3.0, FDR < 0.05) formally identified 23 bacterial, 2 viral, and 7 fungal taxa as tissue-discriminatory biomarkers between the duodenum and colon (Figure 3p–r). Among bacteria, 11 genera were significantly enriched in the duodenum, including the probiotic-associated taxa *Lactobacillus* (log2FC = 3.14), *Bifidobacterium* (log2FC = 2.69), *Enterococcus* (log2FC = 3.10), and *Streptococcus* (log2FC = 2.42). Twelve genera were significantly enriched in the colon, dominated by the fiber-degrading and short-chain fatty acid (SCFA)-producing taxa *Faecalibacterium* (log2FC = −4.77), *Eubacterium* (log2FC = −4.19), *Roseburia* (log2FC = −4.01), *Prevotella* (log2FC = −3.70), *Clostridium* (log2FC = −3.20), and *Bacteroides* (log2FC = −3.08). The viral LEfSe identified *Mamastrovirus* as a duodenal biomarker and *Circovirus* as a colonic biomarker (Figure 3q). Fungal LEfSe identified five duodenum-enriched taxa (*Fusarium*, *Penicillium*, *Aspergillus*, *Fusarium_virus*, and *Penicillium_virus*) and two colon-enriched taxa (*Saccharomyces* and *Cryptococcus*) (Figure 3r). The pronounced tissue specificity of microbial biomarkers underscores the fundamental ecological differentiation between the small and large intestinal niches and provides a reference framework for site-specific microbiome modulation strategies in Tibetan pig production.

### 3.4. Colon Metabolomic Profiling

**Figure 4:**
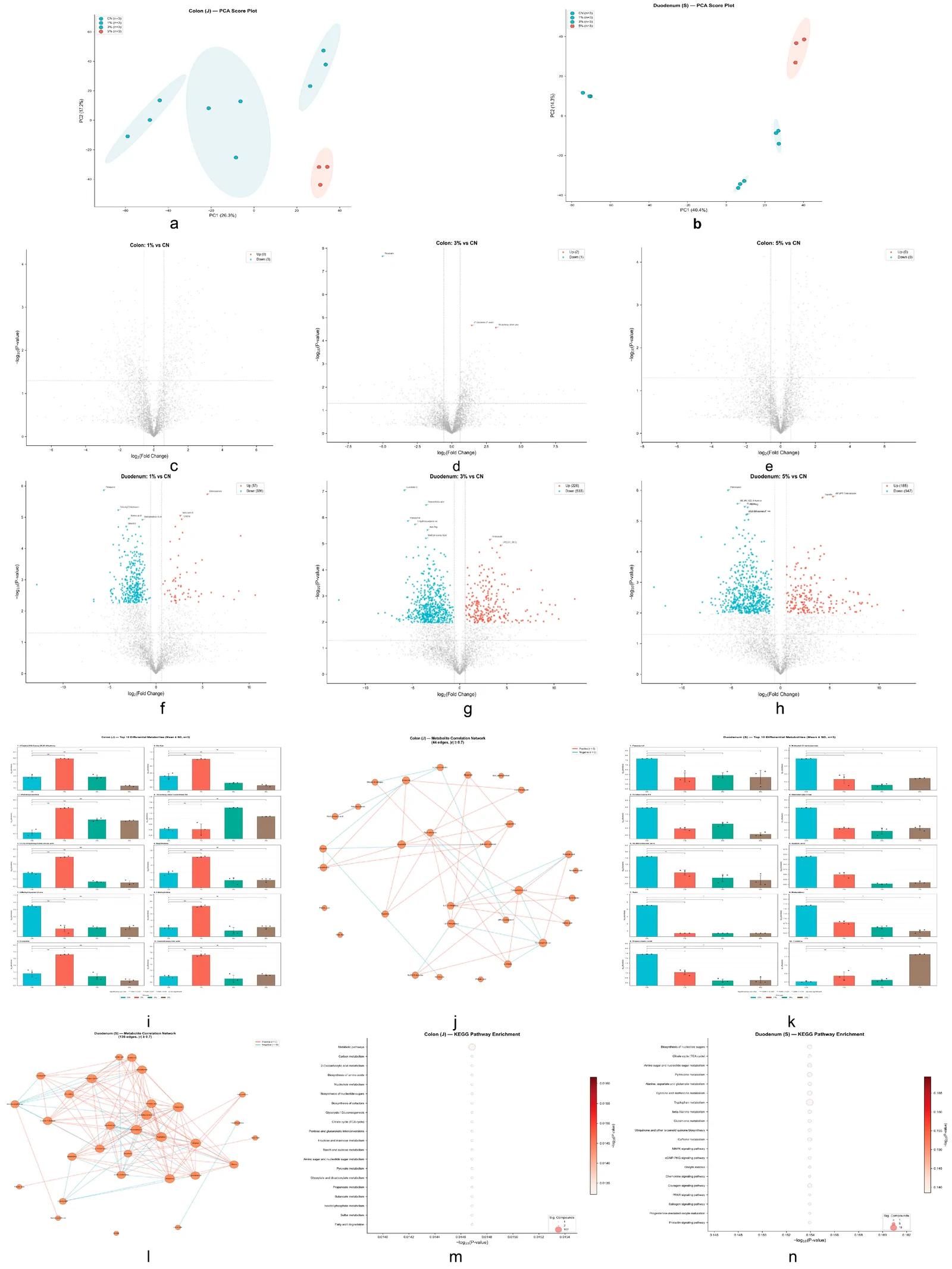
Comprehensive metabolomic profiling of the colon in Tibetan pigs fed with different additive levels. (**a**) Colon (J) PCA Score Plot; (**b**) Duodenum (S) PCA Score Plot; (**c**) Colon: 1% vs. CN (Volcano plot, showing Up and Down regulated metabolites); (**d**) Colon: 3% vs. CN (Volcano plot); (**e**) Colon: 5% vs. CN (Volcano plot); (**f**) Duodenum: 1% vs. CN (Volcano plot); (**g**) Duodenum: 3% vs. CN (Volcano plot); (**h**) Duodenum: 5% vs. CN (Volcano plot); (**i**) Colon (J) Top 10 Differential Metabolites (Mean ± SD, *n* = 3); (**j**) Colon (J) Metabolite Correlation Network (44 edges); (**k**) Duodenum (S) Top 10 Differential Metabolites (Mean ± SD, *n* = 3); (**l**) Duodenum (S) Metabolite Correlation Network (159 edges); (**m**) Colon (J) KEGG Pathway Enrichment; (**n**) Duodenum (S) KEGG Pathway Enrichment.

Untargeted UHPLC-MS/MS metabolomics of colon tissue samples detected 3553–3560 metabolites in total across all samples. In the multi-group comparison (J1 vs. J2 vs. J3 vs. J4), 2180 metabolites were identified as significantly different by multi-group ANOVA (FDR < 0.05). The marked disparity between the high number of multi-group significant metabolites (2180) and the limited pairwise significant metabolites (0–3) reflects the inherent statistical difference between the multi-group ANOVA, which detects any between-group variation across all four groups simultaneously, and the stricter pairwise *t*-tests with FDR correction, which require larger fold changes and lower variance to reach significance in individual dose-level comparisons versus control, with FDR values ranging from 1.02 × 10^−7^ to 1.0 and log(fold change) values spanning from −12.82 to 12.45. PCA revealed distinct metabolic clustering among the four groups, with PC1 and PC2 explaining the major variance components (Figure 4a).

Pairwise comparisons of each additive-supplemented group against the control revealed limited numbers of metabolites reaching the FDR < 0.05 threshold at individual dose levels. Specifically, the 3% additive group (J3) versus control (J1) identified 3 significant differential metabolites: two upregulated (18-carboxy dinor Leukotriene B4, FC = 9.10; 3-Deamino-3-oxonicotianamine, FC = 2.72) and one downregulated (Prostratin, FC = 0.03). In contrast, zero significant differential metabolites were identified in the 1% (J2) versus control (J1) or 5% (J4) versus control (J1) pairwise comparisons (Figure 4b,f).

The top 10 most significant differential metabolites ranked by *p*-value from the multi-group ANOVA included N-Acetyl-D-lactosamine (FDR = 1.02 × 10^−7^), Panaxynol (FDR = 1.02 × 10^−7^), Goshonoside F2 (FDR = 1.47 × 10^−7^), 10-Nitrolinoleic acid (FDR = 1.47 × 10^−7^), Glutaminylproline (FDR = 1.47 × 10^−7^), Azelaic acid (FDR = 1.59 × 10^−7^), Cotinine (FDR = 2.50 × 10^−7^), Metamitron (FDR = 2.50 × 10^−7^), Bisnorcholic acid (FDR = 2.50 × 10^−7^), and Tutin (FDR = 2.50 × 10^−7^) (Figure 4c). Hierarchical clustering of the top 25 differential metabolites based on Z-score-normalized abundance revealed clear segregation among the four dosage groups, consistent with PCA results (Figure 4d).

KEGG pathway enrichment analysis identified the top 15 significantly enriched pathways (Figure 4e), encompassing amino acid metabolism, lipid metabolism, and xenobiotic biodegradation pathways. Notably, the duodenal segment (S groups) showed markedly greater numbers of differential metabolites (S1 vs. S2: 396; S1 vs. S3: 767; S1 vs. S4: 741), indicating a stronger metabolic responsiveness of the duodenum compared to the colon under the same dietary intervention.

### 3.5. Microbiome–Pathology Associations

**Figure 5:**
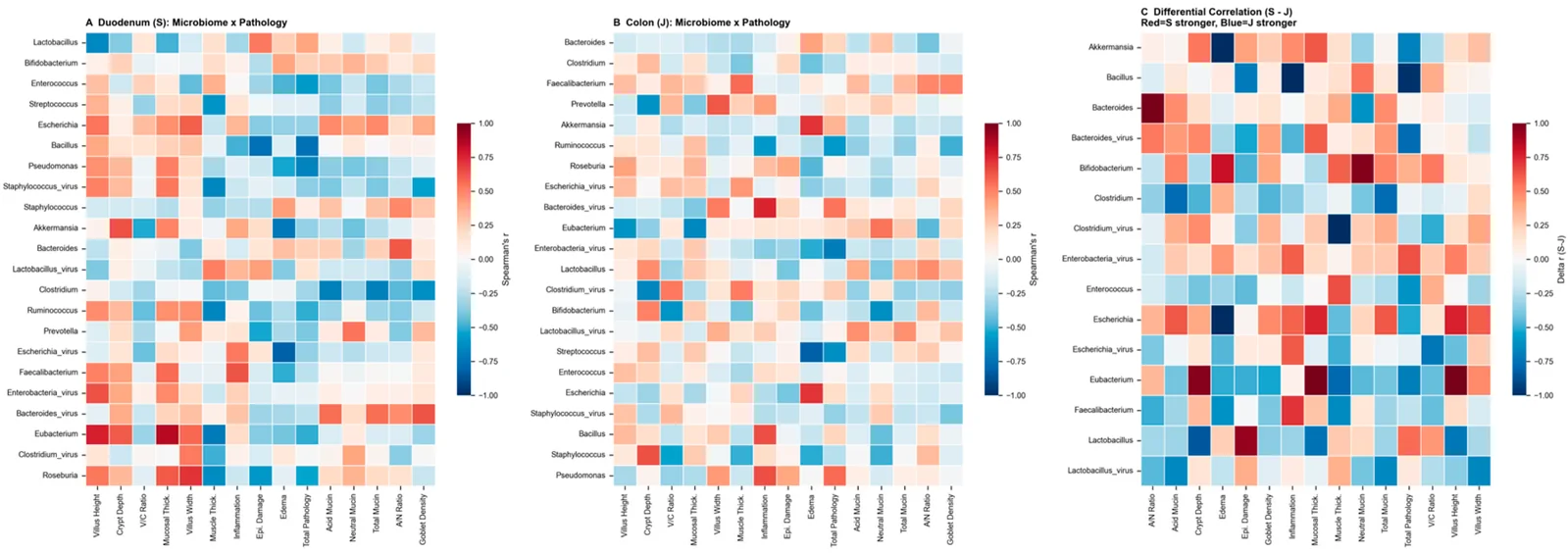
Site-stratified multi-omics integrative correlation analysis of the duodenum and colon. (**A**) Duodenum (S site) microbe–pathology correlation heatmap showing 79 significant pairs (FDR < 0.3, |ρ| > 0.4). Rows: 22 microbial taxa (16 bacterial genera, blue; 6 viral families, red). Columns: 17 histopathological metrics from H&E (12) and AB-PAS (5) staining. Cell color and circle size are proportional to the Spearman correlation coefficient (ρ): red, positive; blue, negative. (**B**) Colon (J site) microbe–pathology correlation heatmap showing 38 significant pairs involving 19 microbial taxa (14 bacterial genera and 5 viral families). (**C**) Differential correlation heatmap displaying Δρ = ρ-S-ρ-J for each microbe–pathology pair. Positive Δρ (red) indicates a stronger positive (or weaker negative) association in the duodenum; negative Δρ (blue) indicates the converse. Gray cells denote pairs not reaching significance at either site.

In the duodenum (S site), 79 significant microbiome–pathology associations were identified (FDR < 0.3, |ρ| > 0.4), comprising 61 bacterial (77.2%) and 18 viral (22.8%) links (Figure 5A). In the colon (J site), 38 significant associations were identified (28 bacterial, 73.7%; 10 viral, 26.3%; Figure 5B). No fungal taxa reached significance at either intestinal site. Across both sites combined, a total of 117 significant microbe–pathology (MB-Path) pairs were detected, encompassing 17 histopathological metrics and 29 unique microbial taxa with significant associations (Bacteria: 22 genera; Viruses: 7 families). Strikingly, every MB-Path association was exclusive to a single anatomical site: 79 pairs (67.5%) were detected solely in the duodenum and 38 pairs (32.5%) solely in the colon, with zero pairs reaching significance at both sites (Figure 5C). This complete site exclusivity underscores a fundamental divergence in how microbial taxa associate with host pathology along the gastrointestinal tract.

Stratification of the duodenal associations by pathology category revealed that morphological parameters (villus height, crypt depth, V/C ratio, villus width, mucosal thickness, and muscle thickness) accounted for the largest share (*n* = 40, 50.6%), followed by inflammation and pathology scores (inflammation, epithelial damage, edema, and total pathology; *n* = 22, 27.8%) and mucin-related metrics (blue mucin, PAS mucin, total mucin, acid/neutral ratio, and goblet cell density; *n* = 17, 21.5%). Eubacterium showed the strongest positive correlations with mucosal morphological parameters: Eubacterium abundance was robustly positively correlated with mucosal thickness (ρ = +0.853, raw *p* = 0.0013, FDR = 0.052) and villus height (ρ = +0.769, raw *p* = 0.0034, FDR = 0.088). As a canonical SCFA-producing genus, the strong association between Eubacterium and healthy mucosal architecture is likely mediated, in part, through butyrate-driven promotion of enterocyte proliferation and intestinal barrier integrity. Roseburia, another well-established butyrate producer, was positively correlated with villus width (ρ = +0.713, raw *p* = 0.0051, FDR = 0.106) and mucosal thickness (ρ = +0.615, raw *p* = 0.0067, FDR = 0.118), coupled with a negative correlation with muscle thickness (ρ = −0.630, raw *p* = 0.0069, FDR = 0.118).

In the duodenum, the most pronounced negative associations wereEscherichia phagewith edema score (ρ = −0.816, raw *p* = 0.0011, FDR = 0.052), Bacillus with epithelial damage (ρ = −0.753, raw *p* = 0.0031, FDR = 0.088) and total pathology score (ρ = −0.752, raw *p* = 0.0032, FDR = 0.088), Akkermansia with edema (ρ = −0.726, raw *p* = 0.0048, FDR = 0.106), and Clostridiumwith mucin metrics—blue mucin (ρ = −0.685, raw *p* = 0.0078, FDR = 0.118), total mucin (ρ = −0.685, raw *p* = 0.0078, FDR = 0.118), and goblet cell density (ρ = −0.623, raw *p* = 0.0089, FDR = 0.118). Among viral taxa, Staphylococcus phage was positively correlated with villus height (ρ = +0.503, raw *p* = 0.0142) and mucosal thickness (ρ = +0.538, raw *p* = 0.0108), while Enterobacteria phagewas positively correlated with villus height (ρ = +0.636, raw *p* = 0.0071, FDR = 0.118), indicating that bacteriophages participate in the microbe–host pathology network alongside their bacterial hosts.

In the colon, the correlation landscape was qualitatively distinct (Figure 5B). Streptococcus exhibited the strongest negative association with edema score (ρ = −0.819, raw *p* = 0.0010, FDR = 0.052), while Bacteroides phage was positively correlated with inflammation score (ρ = +0.753, raw *p* = 0.0030, FDR = 0.088) and total pathology score (ρ = +0.542, raw *p* = 0.0112). Escherichia showed a colon-specific positive association with edema (ρ = +0.717, raw *p* = 0.0047, FDR = 0.106), in contrast to its non-significant edema association in the duodenum. Pseudomonas was positively correlated with inflammation in the colon (ρ = +0.641, raw *p* = 0.0075, FDR = 0.118), and Bifidobacterium demonstrated a significant negative correlation with PAS mucin content (ρ = −0.625, raw *p* = 0.0085, FDR = 0.118) exclusive to the colon. Enterobacteria phage showed protective associations in the colon (total pathology score: ρ = −0.702, FDR = 0.107; edema: ρ = −0.512).

In the duodenum (S site), 211 strong microbiome–metabolite correlations were identified (|ρ| > 0.5; Bacteria = 195, 92.4%; Viruses = 16, 7.6%) involving 22 microbial taxa (16 bacterial genera and 6 viral families) and 97 unique metabolites. These associations encompassed diverse metabolic pathways, including amino acid metabolism (tryptophan, phenylalanine, and tyramine pathways), lipid metabolism (bile acids, fatty acids), and nucleotide metabolism, indicating that the duodenal microbiota is extensively functionally coupled to host physiology through metabolic intermediates. *Lactobacillus* exhibited the most extensive metabolomic associations (52 pairs), followed by *Enterococcus* (48 pairs) and *Ruminococcus* (31 pairs), positioning these genera as central hubs of small-intestinal microbe–metabolite crosstalk. Among the strongest correlations, *Pseudomonas* and *Ruminococcus* each showed robust positive associations with metabolite ME0194137 (ρ = 0.89 and ρ = 0.87, respectively), *Ruminococcus* with ME0110971 (ρ = 0.85), and *Lactobacillus* showed a strong negative correlation with ME0103073 (ρ = −0.83). Viruses contributed 16 significant metabolomic associations (7.6% of MB-Metab pairs), highlighting a non-negligible role of the gut virome in modulating the small-intestinal metabolome.

The differential correlation analysis (Δρ = ρ_S − ρ_J) formally quantified site-specific divergence (Figure 5C) and confirmed that microbial taxa displayed fundamentally different—and sometimes opposing—pathological associations across intestinal compartments. Among the ten taxa detected at both sites, several exhibited directionality shifts: Prevotella was positively correlated with PAS mucin in the duodenum (ρ = +0.545, raw *p* = 0.0099) but was negatively correlated with crypt depth in the colon (ρ = −0.608, raw *p* = 0.0087); Faecalibacterium showed a positive association with inflammation in the duodenum (ρ = +0.628, raw *p* = 0.0079, FDR = 0.118) but was associated with muscle thickness and goblet cell density in the colon; and Escherichia phage shifted from a strong negative edema association in the duodenum (ρ = −0.816, raw *p* = 0.0011, FDR = 0.052) to non-significance in the colon. Collectively, these results reveal that microbe–pathology relationships in the Tibetan pig gut are profoundly site-dependent, with the duodenum serving as the dominant hub of microbe–host crosstalk (79 vs. 38 pairs) and 100% of significant MB-Path associations being site-exclusive.

## 4. Discussion

In this study, we employed an integrated multi-omics approach combining growth performance evaluation, computational histopathology, metatranscriptomic profiling (bacteria, viruses, and fungi), untargeted metabolomics, and multi-omics correlation network analysis to systematically evaluate the effects of a novel antibiotic alternative additive at three dietary inclusion levels (1%, 3%, and 5%) in Tibetan pigs. Our findings collectively suggest that the 3% supplementation level provides the most favorable overall response in terms of growth performance and metabolic modulation without compromising intestinal structural integrity [24].

One-way ANOVA revealed a significant overall treatment effect on ADG (*p* = 0.027) and FCR (*p* = 0.023), with the 3% group showing the most favorable values, consistent across multiple metrics. The 3% additive group exhibited the highest ADG (0.197 kg/d, +33.1% relative to control) and the lowest FCR, while the 1% and 5% groups showed intermediate responses. This non-linear dose–response pattern—where the middle dose yields the greatest benefit—has been observed in other studies of phytogenic feed additives and probiotics in swine [25,26] and may reflect an optimal threshold for bioactive compound efficacy, beyond which excessive concentrations may impose metabolic costs or alter gut ecosystem dynamics in less favorable ways. The 5% group’s numerically higher ADFI without a proportional gain in ADG may reflect several mechanisms: metabolic burden from excessive fermentation products requiring hepatic detoxification, alterations in nutrient digestibility due to supraphysiological levels of organic acids altering intestinal pH and enzyme activity, and potential microbial dysbiosis at high inclusion levels, as suggested by the non-significant metabolic shifts in the 5% vs. control pairwise metabolomic comparison. The 3% level appears to represent an optimal threshold balancing bioactive compound efficacy with metabolic cost. The 5% group’s numerically higher ADFI without a proportional gain in ADG (FCR = 3.05) suggests that supra-optimal additive levels may reduce feed efficiency, possibly due to increased metabolic demand for detoxification or alterations in nutrient digestibility. Future studies with larger sample sizes (n ≥ 15–20 per group) and longer feeding durations are required to achieve sufficient statistical power to confirm these dose–response relationships [25,26]. A critical finding of this study is that the antibiotic alternative additive did not induce any significant histopathological alterations in the intestinal epithelium at any inclusion level. In both the jejunum and duodenum, H&E-based morphometric parameters—including villus height, crypt depth, V/C ratio, and muscle thickness—were comparable across all groups, as were inflammation and total pathology scores. This is an important preliminary safety consideration, as certain phytogenic compounds at high doses have been reported to cause mild epithelial irritation or villus blunting [26]. The preservation of normal villus architecture across all treatment groups supports the gastrointestinal tolerability of this additive at the tested inclusion levels [26,27].

The trend-level decrease in jejunal goblet cell density observed in the treated groups (CN: 661 vs. treated: 82–103 cells/mm^2^; KW *p* = 0.086) warrants careful interpretation. Goblet cell density is dynamically regulated by microbial signals, dietary factors, and immune status [27]. A reduction could reflect either decreased mucin demand due to reduced microbial challenge (a potentially beneficial adaptation) or impaired mucus barrier function. The concomitant numerical decrease in acidic mucin in treated groups (CN: 7.82% vs. 1.77–3.85%; KW *p* = 0.108) suggests a shift in mucin composition that merits further investigation using mucin glycoprofiling and functional barrier assays. In the duodenum, the dose-dependent increase in acidic mucin from 1.21% (CN) to 7.59% (5%), though not statistically significant, is consistent with the known role of dietary fiber and SCFAs in stimulating mucin production [28].

The bacterial community structure observed in Tibetan pigs exhibited a pronounced duodenal-colonic dichotomy (Figure 3). The duodenal bacteriome was dominated by *Lactobacillus* (~35%), *Bifidobacterium* (~18%), *Streptococcus* (~12%), and *Enterococcus* (~10%), genera typically associated with small-intestinal aerobic or facultative anaerobic niches. In contrast, the colonic bacteriome was enriched in obligate anaerobic and fiber-degrading, SCFA-producing taxa, including *Bacteroides* (~16%), *Faecalibacterium* (~12%), *Clostridium* (~11%), *Prevotella* (~9%), *Roseburia* (~6%), *Ruminococcus* (~7%), *Eubacterium* (~4%), and *Akkermansia* (~7%). This spatial organization—*Lactobacillaceae* dominant in the proximal gut and *Bacteroidaceae-Ruminococcaceae*-dominant in the distal gut—differs from the composition typically reported in commercial pig breeds [29], which shows a more uniform *Lactobacillus-Prevotella-Clostridium* dominance pattern [29,30], and may reflect breed-specific microbiome adaptations to the high-altitude plateau environment. LEfSe analysis confirmed that 11 bacterial genera were significantly enriched in the duodenum and 12 in the colon (LDA > 3.0, FDR < 0.05), with the most discriminatory taxa being *Faecalibacterium* (log2FC = −4.77, J-enriched), *Eubacterium* (log2FC = −4.19, J-enriched), Roseburia (log2FC = −4.01, J-enriched), and *Lactobacillus* (log2FC = 3.14, S-enriched). The most robust microbiome–pathology associations involved well-characterized SCFA-producing bacteria. *Eubacterium* and *Roseburia*, both known for their butyrate-producing capacity, were strongly positively correlated with mucosal thickness (ρ = +0.853 and ρ = +0.615, respectively), villus height (ρ = +0.769 and ρ = +0.538), and villus width (ρ = +0.573 and ρ = +0.713), indicating a robust and reproducible association between SCFA-producing bacteria and healthy mucosal architecture. Butyrate serves as the primary energy substrate for colonocytes, promotes tight junction protein expression, and exerts anti-inflammatory effects through histone deacetylase inhibition and G-protein-coupled receptor signaling [29,30,31,32].

A novel contribution of our study is the inclusion of the virome and mycobiome in the analysis, which are often overlooked in livestock microbiome research. The gut virome was dominated by *Pisuviricota* and *Cressdnaviricota*, and LEfSe analysis identified *Mamastrovirus* as a duodenal biomarker and *Circovirus* as a colonic biomarker (Figure 3q). The fungal community was dominated by *Ascomycota* (83–87%), with *Candida*, *Aspergillus*, and *Penicillium* as the most abundant genera. Seven fungal genera were identified as tissue-discriminatory biomarkers, including duodenum-enriched *Fusarium*, *Penicillium*, and *Aspergillus*, and colon-enriched *Saccharomyces* and *Cryptococcus* (Figure 3r). The positive correlation of *Bacteroides phage* with inflammation scores and of *Enterobacteria phage* with villus height highlights the tripartite regulatory interactions among phages, their bacterial hosts, and the host immune system [33]. The tissue-specific distribution of bacteriophages—with phages targeting small-intestine-enriched genera (*Staphylococcus phage*, *Lactobacillus phage*) more abundant in the duodenum, and phages targeting colon-enriched genera (*Escherichia phage*, *Enterobacteria phage*, *Bacteroides phage*, *Clostridium phage*) more abundant in the colon—further suggests active phage-bacterium co-occurrence dynamics along the gastrointestinal tract [34].

The multi-omics network exhibiting a core–periphery architecture, with *Eubacterium*, *Lactobacillus*, *Enterococcus*, and *Escherichia* as highly connected hub nodes, provides an exploratory, systems-level framework (based on group-level correlations, *n* = 4 groups) for generating hypotheses about how the gut microbiome may influence host phenotypes. These correlations represent statistical associations, and causal relationships remain to be established through targeted mechanistic studies. The duodenal-centric network topology—anchored by genera that dominate the small-intestine ecosystem (*Lactobacillus*, *Enterococcus*) and interfacing with colon-enriched butyrate producers (*Eubacterium*, *Roseburia*)—highlights the interconnected nature of microbial metabolism across intestinal segments. The identification of these keystone taxa suggests that targeted modulation of these microbial populations may represent an effective strategy for improving intestinal health in Tibetan pigs.

Several limitations should be acknowledged. First, the small sample size for histological and multi-omics analyses (*n* = 3 per group) is a critical limitation. With *n* = 3, the minimum achievable two-sided Mann–Whitney U *p*-value is 0.10, which means that only trend-level significance can be reported. The risk of Type II error (failure to detect true biological differences) is substantial, and the microbial and histological results must be interpreted as exploratory. Future studies should employ a priori power calculations to determine adequate sample sizes (n ≥ 5–6 per group for histology; n ≥ 8–10 per group for multi-omics) based on the effect sizes observed in this study. Second, the anatomical mismatch between histological sampling (jejunum and duodenum) and metatranscriptomic/metabolomic sampling (duodenum and colon) limits direct tissue-level integration of microbiome and pathology data. Specifically, jejunal histology cannot be directly correlated with colonic metatranscriptomics, and the multi-omics correlation network was constructed at the group level (*n* = 4 groups per site) rather than at the individual animal level, which severely reduces the degrees of freedom and increases the risk of spurious correlations. The network results should therefore be interpreted as hypothesis-generating rather than as robust individual-level biological associations. Third, the multi-omics integration was performed at the treatment-group level (*n* = 4) rather than at the individual animal level, which further limits statistical robustness. Future studies should collect fully matched samples from all intestinal segments for each analytical modality to enable individual-level multi-omics integration. Fourth, the 21-day feeding period may not capture long-term adaptive changes; extended trials of 6–12 weeks are recommended. First, the relatively small sample size for histological analyses (*n* = 3 per group) limited statistical power; future studies should aim for *n* ≥ 5–6 per group. Second, histological sampling (jejunum and duodenum) and metatranscriptomic/metabolomic sampling (duodenum and colon) were not fully matched across intestinal sites, limiting direct tissue-level integration of microbiome and pathology data; future studies should collect matched samples from all intestinal segments for each analytical modality. Fifth, the 21-day feeding period may not capture long-term adaptive changes; extended trials of 6–12 weeks are recommended. Sixth, the metatranscriptomic analysis provided taxonomic but not functional profiling; shotgun metagenomic functional annotation would provide deeper functional insights. Seventh, the multi-omics correlation network identified statistically robust associations that are correlational in nature; causal validation through targeted microbial manipulation is necessary to establish mechanistic links.

## 5. Conclusions

This comprehensive multi-omics study provides exploratory evidence that dietary supplementation with a novel antibiotic alternative additive at graded inclusion levels (1%, 3%, and 5%) in Tibetan pigs results in a non-linear dose–response. Growth performance showed numerical improvement at the 3% level, and intestinal epithelial structural integrity was preserved across all treatment levels, supporting gastrointestinal safety. The gut microbial ecosystem was modulated in a site-dependent manner, with LEfSe analysis identifying 32 tissue-discriminatory biomarker taxa between the duodenum and colon. Dose-dependent metabolomic reprogramming was observed, with the most pronounced metabolic shift at the 3% level. Furthermore, the multi-omics correlation network revealed a core–periphery architecture in which beneficial SCFA-producing bacteria serve as keystone hubs positively associated with mucosal health and villus architecture, whereas opportunistic pathogens and specific bacteriophages were linked to inflammatory indices and tissue pathology. Collectively, these exploratory findings suggest that this antibiotic alternative additive, particularly at the 3% inclusion level, warrants further investigation in larger-scale, longer-term confirmatory studies to validate its potential for improving production efficiency and intestinal health in Tibetan pig farming. Future large-scale, long-term studies with functional metatranscriptomic and causal validation approaches are warranted.

## Figures and Tables

**Table 1:** Effects of dietary antibiotic alternative additive on growth performance of Tibetan pigs (mean ± SEM).

Item	CN (Control)	1% Additive	3% Additive	5% Additive	*p*-Value
No. of animals	10	10	10	10	
IBW, kg	8.26 ± 0.34	7.97 ± 0.40	8.71 ± 0.36	7.72 ± 0.49	0.347
FBW, kg	11.36 ± 0.58	11.59 ± 0.78	12.85 ± 0.36	11.47 ± 0.68	0.293
ADG, kg/d	0.148 ± 0.016 ^c^	0.172 ± 0.022 ^b^	0.197 ± 0.017 ^a^	0.179 ± 0.012 ^b^	0.027
ADFI, kg/d	0.476 ± 0.018	0.476 ± 0.026	0.476 ± 0.016	0.529 ± 0.031	0.317
FCR	3.59 ± 0.39 ^a^	3.24 ± 0.43 ^b^	2.61 ± 0.25 ^d^	3.05 ± 0.22 ^c^	0.023

IBW, initial body weight; FBW, final body weight; ADG, average daily gain; ADFI, average daily feed intake; FCR, feed conversion ratio (ADFI/ADG). *p*-values were obtained from one-way ANOVA with Tukey HSD post hoc test. Values within a row without common superscript letters differ significantly (*p* < 0.05). Feeding trial duration: 21 days (4–25 December 2025). *n* = 10 per group.

**Table 2:** Jejunal histomorphometric and mucin parameters of Tibetan pigs fed different levels of antibiotic alternative additive (mean ± SEM, *n* = 3/group).

Metric	CN	1%	3%	5%	KW *p*	Sig.
Villus height (rel.)	0.279 ± 0.043	0.279 ± 0.012	0.261 ± 0.037	0.318 ± 0.074	0.838	n.s.
Crypt depth (rel.)	0.339 ± 0.091	0.320 ± 0.026	0.291 ± 0.027	0.333 ± 0.016	0.644	n.s.
V/C ratio	0.938 ± 0.220	0.889 ± 0.107	0.924 ± 0.180	0.975 ± 0.266	0.999	n.s.
Muscle thickness (rel.)	0.026 ± 0.020	0.027 ± 0.019	0.031 ± 0.016	0.015 ± 0.012	0.863	n.s.
Inflammation score	3.00 ± 0.00	2.67 ± 0.33	3.00 ± 0.00	2.33 ± 0.33	0.214	n.s.
Total pathology score	5.67 ± 0.33	4.67 ± 0.33	5.67 ± 0.33	4.67 ± 0.33	0.112	n.s.
Acidic mucin AB+ (%)	7.82 ± 1.74	1.80 ± 1.38	1.77 ± 1.23	3.85 ± 1.57	0.108	n.s.
Neutral mucin PAS+ (%)	0.079 ± 0.074	0.047 ± 0.041	0.103 ± 0.050	0.183 ± 0.163	0.731	n.s.
Total mucin (%)	7.90 ± 1.82	1.85 ± 1.42	1.87 ± 1.27	4.03 ± 1.52	0.108	n.s.
Goblet cell density (/mm^2^)	660.9 ± 230.6	103.4 ± 99.8	82.0 ± 77.7	88.7 ± 57.8	0.086	† (trend)
Acid/Neutral ratio	2153 ± 1815	150 ± 117	19.0 ± 6.3	222 ± 190	0.129	n.s.

rel., normalized relative to image height; n.s., not significant; KW, Kruskal–Wallis test. † *p* < 0.10 (trend). Minimum achievable two-sided MWU *p*-value with *n* = 3/group = 0.10.

**Table 3:** Duodenal histomorphometric and mucin parameters of Tibetan pigs fed different levels of antibiotic alternative additive (mean ± SEM, *n* = 3/group).

Metric	CN	1%	3%	5%	KW *p*	Sig.
Villus height (rel.)	0.250 ± 0.055	0.326 ± 0.063	0.228 ± 0.041	0.270 ± 0.014	0.764	n.s.
Crypt depth (rel.)	0.388 ± 0.058	0.406 ± 0.099	0.373 ± 0.080	0.393 ± 0.050	0.988	n.s.
V/C ratio	0.630 ± 0.071	0.947 ± 0.373	0.629 ± 0.090	0.704 ± 0.073	0.838	n.s.
Muscle thickness (rel.)	0.046 ± 0.017	0.036 ± 0.033	0.030 ± 0.027	0.044 ± 0.003	0.780	n.s.
Inflammation score	2.67 ± 0.33	2.33 ± 0.33	2.67 ± 0.33	2.33 ± 0.33	0.748	n.s.
Total pathology score	4.67 ± 0.33	3.67 ± 0.33	5.33 ± 0.67	5.00 ± 0.00	0.122	n.s.
Acidic mucin AB+ (%)	1.21 ± 0.62	0.58 ± 0.16	3.04 ± 1.73	7.59 ± 3.52	0.497	n.s.
Neutral mucin PAS+ (%)	0.112 ± 0.076	0.066 ± 0.014	0.037 ± 0.011	0.096 ± 0.032	0.557	n.s.
Total mucin (%)	1.32 ± 0.63	0.65 ± 0.17	3.08 ± 1.73	7.68 ± 3.55	0.497	n.s.
Goblet cell density (/mm^2^)	33.0 ± 19.4	5.4 ± 3.3	170.2 ± 102.2	22.1 ± 13.3	0.580	n.s.
Acid/Neutral ratio	23.7 ± 9.9	8.4 ± 1.4	115.4 ± 89.8	64.6 ± 23.8	0.361	n.s.

rel., normalized relative to image height; n.s., not significant; KW, Kruskal–Wallis test.

## Data Availability

The data presented in this study are openly available in the Genome Sequence Archive (Genomics, Proteomics & Bioinformatics 2025) in National Genomics Data Center (Nucleic Acids Res 2026), China National Center for Bioinformation/Beijing Institute of Genomics, Chinese Academy of Sciences (GSA: CRA075370) that are publicly accessible at https://ngdc.cncb.ac.cn/gsa, accessed on 9 July 2026.

## Supplementary Materials

The following supporting information can be downloaded at: https://www.mdpi.com/article/10.3390/vetsci13080826/s1, Table S1: Diet composition and nutrient levels for Tibetan pigs aged 90–120 days (air-dry basis).
