# Supplementary material for: Effects of an Antibiotic Alternative Additive on Growth Performance, Intestinal Histomorphology, Multi-Omics Profiles, and Microbiome–Host Interactions in Tibetan Pigs
Source: Vet Sci. 2026 Aug 18;13(8):826. doi: 10.3390/vetsci13080826 (PMC13517117; doi:10.3390/vetsci13080826)
Supplement: Supplementary file 1 [file vetsci-13-00826-s001.zip › vetsci-4440999-supplementary.pdf]

Table S1. Diet composition and nutrient levels for Tibetan pigs aged 90–120 days (air-dry basis)

Items	Content
Corn (%)	62.00
Soybean meal (%)	18.00
Wheat bran (%)	8.00
Rapeseed meal (%)	4.00
Alfalfa meal (%)	3.00
Fish meal (%)	1.50
Limestone (%)	1.20
$CaHPO_4$ (%)	0.90
NaCl (%)	0.40
L-Lys·HCl (%)	0.30
DL-Met (%)	0.10
Premix[1)] (%)	0.60
Total	100.00
Nutrient levels[2)]	
DE ($MJ \cdot kg^{-1}$)	12.55
CP (%)	16.50
CF (%)	4.20
EE (%)	3.10
Ca (%)	0.75
TP (%)	0.58
AP (%)	0.32
Lys (%)	0.85
Met (%)	0.28
Met+Cys (%)	0.55
Thr (%)	0.56
Trp (%)	0.15

Note：

[1] Premix provided the following per kg of diet: VA 5 500 IU, VD_3 550 IU, VE 30 IU, VK_3 2.5 mg, VB_1 1.8 mg, VB_2 4.5 mg, VB_6 2.5 mg, VB_{12} 20 μg, nicotinic acid 25 mg, pantothenic acid 15 mg, folic acid 0.5 mg, biotin 0.15 mg, choline 400 mg. [2] Nutrient levels are calculated values. [3] Trace mineral supplementation (added via premix, $mg \cdot kg^{-1}$ diet)：Fe 100 ($FeSO_4 \cdot H_2O$), Cu 10 ($CuSO_4 \cdot 5H_2O$), Zn 80 ($ZnSO_4 \cdot H_2O$), Mn 40 ($MnSO_4 \cdot H_2O$), I 0.50 [$Ca(IO_3)_2$], Se 0.30 (Na_2SeO_3), Co 0.20 ($CoCl_2 \cdot 6H_2O$).

Note：Trace mineral levels refer to NRC (2012) and Chinese Feeding Standard for Swine (NY/T 65-2004), adjusted for Tibetan pig high-altitude feeding conditions.
